# Data-Driven Discovery of Immune Contexture Biomarkers

**DOI:** 10.3389/fonc.2018.00627

**Published:** 2018-12-18

**Authors:** Lars Ole Schwen, Emilia Andersson, Konstanty Korski, Nick Weiss, Sabrina Haase, Fabien Gaire, Horst K. Hahn, André Homeyer, Oliver Grimm

**Affiliations:** ^1^Fraunhofer Institut für Bildgestützte Medizin, Bremen, Germany; ^2^Pathology and Tissue Analytics, Pharma Research and Early Development, Roche Innovation Center Munich, Penzberg, Germany; ^3^Fraunhofer Institut für Bildgestützte Medizin, Lübeck, Germany

**Keywords:** biomarker discovery, cell-to-cell distances, colorectal cancer, digital biomarkers, immune contexture, spatial heterogeneity, tumor microenvironment, whole-slide image analysis

## Abstract

**Background:** Features characterizing the immune contexture (IC) in the tumor microenvironment can be prognostic and predictive biomarkers. Identifying novel biomarkers can be challenging due to complex interactions between immune and tumor cells and the abundance of possible features.

**Methods:** We describe an approach for the data-driven identification of IC biomarkers. For this purpose, we provide mathematical definitions of different feature classes, based on cell densities, cell-to-cell distances, and spatial heterogeneity thereof. Candidate biomarkers are ranked according to their potential for the predictive stratification of patients.

**Results:** We evaluated the approach on a dataset of colorectal cancer patients with variable amounts of microsatellite instability. The most promising features that can be explored as biomarkers were based on cell-to-cell distances and spatial heterogeneity. Both the tumor and non-tumor compartments yielded features that were potentially predictive for therapy response and point in direction of further exploration.

**Conclusion:** The data-driven approach simplifies the identification of promising IC biomarker candidates. Researchers can take guidance from the described approach to accelerate their biomarker research.

## 1. Introduction

### 1.1. Motivation

One of the main contributors to cancer progression is an insufficient antitumor immune response, either because of immunosuppression by the tumor or absent antitumor immunity. The tumor microenvironment is the main stage of the antitumor immune response and, therefore, reveals reasons for its insufficiency (1).

In the tumor microenvironment, a multitude of different immune cells interact with the tumor cells in complex ways. Some immune cells, such as CD8+ T cells, are directly cytotoxic to tumor cells. Other immune cells, such as CD4+ helper T cells or FOXP3+ regulatory T cells, or even tumor cells themselves stimulate or suppress the immune response (1).

Three areas of the tumor microenvironment are of particular interest: the tumor cell compartment, the tumor-associated stroma, and the invasive margin, consisting of the border between the tumor lesion and the surrounding tissue. Immune cell infiltrates in the tumor cell compartment indicate an active immune response, whereas immune cell infiltrates in the invasive margin or only in the intratumoral stroma (but not in the tumor cell compartment) indicate immunosuppression by the tumor. Missing immune cells in all compartments indicate the absence of antitumor immunity (1).

The density and spatial organization of the immune infiltrate in the tumor microenvironment, also called the immune contexture (IC), often correlates with clinical outcome (2, 3). This information is therefore used as a prognostic biomarker.

For this purpose, histological sections of the tumor microenvironment are stained for characteristic antigens of immune and tumor cells. The sections are then digitized and the relevant compartments and cells are annotated with image analysis software (4). From these annotations, a wealth of different features can be computed which describe the density and spatial organization of immune and tumor cells in the tumor microenvironment. In this paper, we use the term “feature” for general characteristics of the immune contexture, and the term “biomarkers” for immune contexture features with prognostic value.

Immunotherapy that resolves immunosuppression by checkpoint blockade has achieved remarkable clinical success. Unfortunately, this success only applies to subgroups of patients. Also, immunotherapy can have severe side effects and be very expensive. IC biomarkers can not only be prognostic but also predictive of immunotherapy response (1). When used as companion diagnostics, such biomarkers can reduce unnecessary suffering and costs.

### 1.2. State of the Art

There is a growing body of literature on IC biomarkers (4). The most simple class quantifies the compartment-specific density of certain immune cells. Prominent examples are given in the publications by Galon et al. that describe a semiquantitative “Immunoscore” derived from the densities of certain immune cells (e.g., CD3/CD8) in the tumor compartment and invasive margin. This score was shown to be a better prognostic factor for colorectal cancer than the established TNM (Tumor, lymph Nodes, Metastases) staging system (5, 6).

Another class of IC biomarkers is derived from distances between different types of cells. The assumption is that cell interactions become apparent through spatial proximity. For instance, Feichtenbeiner et al. found that CD8+ and FOXP3+ immune cells in the tumor compartment positively impact the prognosis of human gastric cancer when their shortest average distance lies between 30 and 110 μm (7). Nagl et al. found that non-random, short distances between FOXP3+ and CD20+ immune cells were associated with unfavorable prognosis in anal squamous cell carcinoma (8).

IC features often vary substantially within the tumor. For example, Krüger et al. report breast cancer cases with substantial overall inflammation but large areas with sparse immune infiltrates. They also report cases with little overall inflammation but focally dense immune infiltrates (9). This has led to the idea to use the intratumoral heterogeneity of the aforementioned features as an immune context feature in itself.

Intratumoral heterogeneity can be quantified by dividing an image into a grid of tiles, and computing statistics about the feature distribution across the tiles. In the analysis of radiological images, this approach has revealed several prognostic biomarkers (10). In pathology, this approach has been used for the assessment of Ki67 expression heterogeneity (11). Besides very recent developments (12), heterogeneity appears to be largely unexplored in the context of IC biomarkers.

Finding novel IC biomarkers is very challenging. On the one hand, there is still little knowledge about the manifold interactions between immune and tumor cells and studies often yield contradictory results (7). This makes it hard to identify biological effects with prognostic or predictive value. On the other hand, IC features can be arbitrarily complex and are often created by combining other IC features, such as densities of different cell types. IC features also often have parameters, such as distance thresholds, that are hard to set in advance. The resulting huge number of possible features makes the selection of promising biomarker candidates challenging.

### 1.3. Contributions

To tackle these challenges, we describe an approach for the data-driven discovery of predictive IC biomarkers. This approach can be used as part of general hypothesis-free biomarker discovery studies such as Beck et al. (13), Harder et al. (14). We thus provide a tool to complement classical, knowledge-driven biomarker research.

We present a systematic overview of possible IC features, based on cell densities, cell-to-cell distances, and spatial heterogeneity, with clear mathematical definitions. We describe a feature selection process to identify biomarker candidates out of a large set of possible features. In this context, we discuss the significance of results obtained from small datasets which are common in immunotherapy trials.

We evaluated the approach on a cohort of 72 colorectal cancer patients with highly variable amounts of microsatellite instability (MSI). MSI colorectal cancers tend to have more abundant immune infiltrates and are more likely to respond to immunotherapy than microsatellite-stable (MSS) cancers (15). Assuming that MSI indicates potential therapy response, we ranked candidate IC features according to their predictive power with regard to MSI.

## 2. Materials and Methods

Our approach for computing quantitative features and the assessment of their predictive power consists of several steps. It is based on object data extracted from fluorescence microscopy, an established procedure that we summarize in section 2.1. After a brief explanation of the biological background of the object data in section 2.2, we describe the computation of a number of quantitative “base” features, including global densities, density ratios, and distance-based features taking into account the possibility of cell-cell interactions (section 2.3). Evaluating these features in tilings of the specimen domain and using techniques from descriptive statistics, we next describe how to quantitatively characterize different aspects of the heterogeneity of said features (section 2.4). Finally, we provide an approach for quantitatively assessing the discriminatory power of the features for binary end points (section 2.5).

The approach presented here is generic and modular in different regards. While we list specific object (cell) types in the Supplementary Data Sheet 1, Section 1.1, the feature computation can be applied to any type of point objects in histology and beyond.

### 2.1. Data Acquisition

#### 2.1.1. Creating Specimens and Imaging

Surgical samples from primary colorectal tumors were procured from Avaden and Indivumed with limited attached clinical data. Surgical samples were collected from consented patients (informed consent) and under approval from the respective Institutional Review Board, National Ethics Committee, or equivalent agency. The samples had been fixed in formalin, embedded in paraffin and archived prior to shipment.

A total of 72 tissue specimens were obtained and histologically processed into 2.5 μm thick sections. Fluorescent immunohistochemistry was used to label Ki67+ cells, CD3+/CD4+ cells and CD3+/CD8+ cells according to the procedure described in Zhang et al. (16). The resulting slides were digitized with a Zeiss Axio Scan.Z1 at 20 × magnification, resulting in a pixel resolution of 325 nm. Additionally, H&E slides used for a reference were digitized with a Ventana iScan HT at 20 × magnification (pixel resolution of 465 nm).

In addition to creating image data for further analysis, the slides were classified as MSI or MSS. For this purpose, slides of the tissue samples were stained and assessed for the presence or absence of four mismatch repair (MMR) proteins, MLH1, MSH2, MSH6, and PMS2. Loss of one or more of the mismatch gene products is highly concordant with DNA-based MSI testing (17–20). Tumors with loss of one or more of the MMR proteins are considered MSI, whereas intact MMR staining is classified as MSS. This resulted in 19 MSI and 53 MSS cases in our dataset.

#### 2.1.2. Image Analysis

The digitized whole-slide images were displayed in a custom image viewer enabling panning, zooming, and rotating images. The entire tissue was detected automatically, the tumor compartment of the tissue and tissue compartment to be excluded from the analysis (e.g., necrotic regions) were drawn and labeled by human experts using said image viewer.

Different cell objects were detected by a custom written machine learning algorithm based on color, intensity, texture, and shape of the objects appearing in the fluorescence images. The algorithmic results were verified by a human expert (pathologist) by visual assessment of the detected objects.

**Figure 1:**
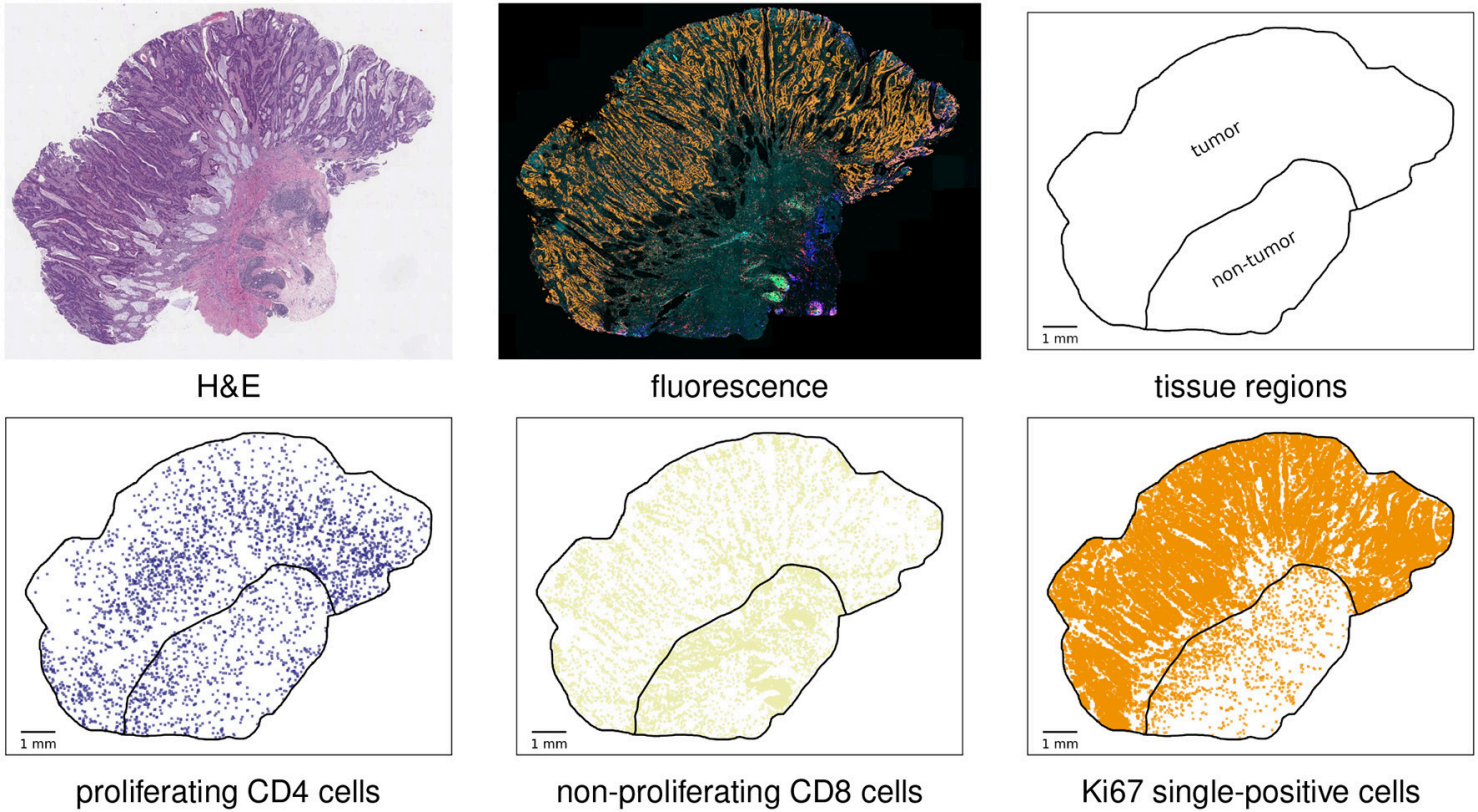
Basic analysis workflow. Top row, from left to right: H&E-stained slide for visual assessment of the specimen; synthetic overlay of the fluorescence images of different cell protein stainings; automatically detected tissue and annotated tumor compartment of the tissue. Bottom row: visualization of three examples of detected cell object types. This case was classified as MSI.

Figure 1 illustrates the data acquisition workflow and shows two object visualizations for an example case.

### 2.2. Data Interpretation

Based on the annotations described in section 2.1.2, three tissue regions are defined: the “entire tissue” (non-excluded, non-necrotic) in the slide, the intersection of the entire tissue and the annotated tumor region as the “tumor compartment,” and the entire tissue without the annotated tumor region as the “non-tumor compartment.”

Moreover, we consider following types of objects,

- Ki67: Ki67 single-positive (i.e., proliferating) cells used as a surrogate for tumor cells;
- CD4^any^, CD4^prolif.^, CD4^non-prolif.^: CD4 immune cells (T helper cells and regulatory T cells), distinguished in proliferating and non-proliferating cells (CD4+ cells that are Ki67+ and Ki67-, respectively); and
- CD8^any^, CD8^prolif.^, CD8^non-prolif.^: CD8 immune cells (cytotoxic T cells/effector cells), distinguished in proliferating and non-proliferating cells (CD8+ cells that are Ki67+ and Ki67-, respectively).

For a more detailed description of the object types, we refer to the Supplementary Data Sheet 1, Section 1.1.

Mathematically, the tissue regions are represented as the interior of polygons denoted as *R*. The cell objects are represented as points denoted as ω, the set of all objects of a certain type is denoted as Ω. For notational convenience, we use the “intersection” Ω∩*R* as shorthand for all objects of type Ω geometrically located in tissue region *R* (entire tissue, tumor compartment, or non-tumor compartment), i.e., mathematically we identify object sets and their types as well as objects and their geometric location.

### 2.3. Global Features

We compute two categories of global features, i.e., features evaluated for the entire tissue or the entire tumor or non-tumor compartment:

- “density-based features” (7, 8) comprising densities of objects and object combinations as well as various ratios of object (combination) counts; and
- “distance-based features” or “features based on cell-to-cell distances” (7, 8).

For these categories, we consider different feature types. We describe each feature type in two steps, first explaining it in text form, then providing a precise and detailed mathematical definition in a separate paragraph.

#### 2.3.1. Density-Based Features

##### 2.3.1.1. Counts

As the most basic features, we count the number of objects of each type in each tissue region, e.g., the number of CD8 immune cells in the non-tumor compartment.

Mathematically, these features are defined as

(1) $$\begin{array}{r} \#\left(\Omega \cap R\right), \end{array}$$

where *R* is either the entire tissue, the tumor compartment, or the non-tumor compartment, and Ω is any of the 10 individual object types or object combinations explained in the Supplementary Data Sheet 1, Section 1.1. Clearly, such counts are dimensionless. By themselves, marker counts are of limited use for characterizing the tissue as they neglect the tissue size, but can be verified easily by counting the respective strings in the input data.

##### 2.3.1.2. Densities

Object densities and densities of object combinations are computed as object counts divided by the area of the respective tissue region, e.g., the density of proliferating CD8 immune cells in the entire slide.

Mathematically, densities are defined as

(2) $$\begin{array}{r} \begin{array}{c} \frac{\#(\Omega \cap^{\text{​}}R)}{\text{area}(R)}, \end{array} \end{array}$$

where, as above, *R* is either the slide, the tumor, or the non-tumor compartment, and Ω is any of the 10 individual object type or object combinations explained in the Supplementary Data Sheet 1, Section 1.1. Region area is measured in millimeters squared, so the densities are given in units 1 per mm^2^.

##### 2.3.1.3. Ratios of cell types

For certain object combinations, we compute the ratios of the respective object counts in the tissue regions, e.g., the ratio of CD4 immune cells and Ki67 single-positive cells in the tumor compartment.

Mathematically, these ratio features are

(3) $$\begin{array}{r} \frac{\#\left(\Omega_{a}\cap R\right)}{\#\left(\Omega_{b}\cap R\right)}, \end{array}$$

where (Ω_*a*_, Ω_*b*_) is one of the 156 distinct object combinations listed in the Supplementary Data Sheet 1, Section 1.1, and *R* is, as above, either the slide, the tumor, or the non-tumor tissue. The counts and thus their ratios are dimensionless. Note that these ratios include the special case of where Ω_*a*_ is a subset of Ω_*b*_, e.g., the ratio of proliferating CD4 over all CD4, which can also be denoted as the “fraction” between 0 and 1 of proliferating CD4 cells.

#### 2.3.2. Distance-Based Features

In order to model limited spatial influence of cells in their neighborhood, we also compute features taking into account minimal or maximal distances of object types to the closest object of a different type (denoted as “reference objects”). Examples include the number of CD4 immune cells no farther than 30 μm from the closest Ki67 single-positive cell, or Ki67 single-positive cells at least 50 μm away from the closest CD4 immune cell.

Mathematically, we distinguish two cases of increasing complexity: single and combined reference object types. The previous examples contain Ki67 single-positive as a single reference object type, and (proliferating and non-proliferating) CD4 immune cells as a combined object type.

##### 2.3.2.1. Cell-to-cell distance criteria

Mathematically, distances to single reference object types can be expressed by equipping the numerator and denominator in Equation (3) with distance criteria. In the simplest case, the count is restricted to objects of type Ω_*a*_ whose distance to the closest object of type Ω_*b*_ (reference objects) is within a given threshold,

(4) $$\begin{array}{r} \begin{array}{c} \#\{\omega_{a}\in \Omega_{a}\cap^{\text{​}}R:\text{dist}(\omega_{a},\Omega_{b})\leq \theta \}, \end{array} \end{array}$$

where we consider thresholds in the range of a few typical cell sizes,

(5) $$\begin{array}{r} \theta \in \left\{15,20,25,30,35,50,100\right\}\mu \text{m}, \end{array}$$

in order to capture interactions of directly adjacent cells as well as, e.g., interactions via cytokines in the vicinity. A restriction to objects with distance above a threshold is obtained by replacing ≤ by > in Equation (4).

##### 2.3.2.2. Adjacency to multiple cell types

Mathematically, distances to combined reference object types (e.g., proliferating and non-proliferating and CD4 immune cells) provide a richer set of features, but are also more complicated to describe. For reference objects in the distance criterion being a combination M of object sets Ω∈M, one can either require that a proliferating *or* a non-proliferating CD4 object need to be closer than θ, or that both a proliferating *and* a non-proliferating object are needed. For the “or” case, this results in features of the form

(6) $$\begin{array}{r} \#\left\{\omega_{a}\in \Omega_{a}\cap R:\vee_{\Omega \in M}\text{dist}\left(\omega_{a},\Omega \right)\leq \theta \right\}. \end{array}$$

For, e.g., $M=\left\{\Omega_{\beta },\Omega_{\gamma },\Omega_{\delta }\right\}$, we here use the notation $\vee_{\Omega \in M}\text{ dist }\left(\omega_{a},\Omega \right)\leq \theta$ as shorthand for

(7) $$\begin{array}{r} (\text{dist}\left(\omega_{a},\Omega_{\beta }\right)\leq \theta )\vee (\text{dist}\left(\omega_{a},\Omega_{\gamma }\right)\leq \theta )\vee (\text{dist}\left(\omega_{a},\Omega_{\delta }\right)\leq \theta ). \end{array}$$

The “and” case, in analogy to Equation (7), uses the notation $\wedge_{\Omega \in M}\text{dist}\left(\omega_{a},\Omega \right)\leq \theta$. Moreover, one can require minimum rather than maximum distances. In total, this translates to four possible distance criteria:

(8) $$\#\{\omega_{a}\in \Omega_{a}\cap R:\left\{\begin{array}{l} \vee_{\Omega \in M}\\ \wedge_{\Omega \in M} \end{array}\right\}\text{ dist }\left(\omega_{a},\Omega \right)\left\{{}_{>}^{\leq }\right\}\theta \}.$$

##### 2.3.2.3. Distance-based rwith the distancesatios

**Figure 2:**
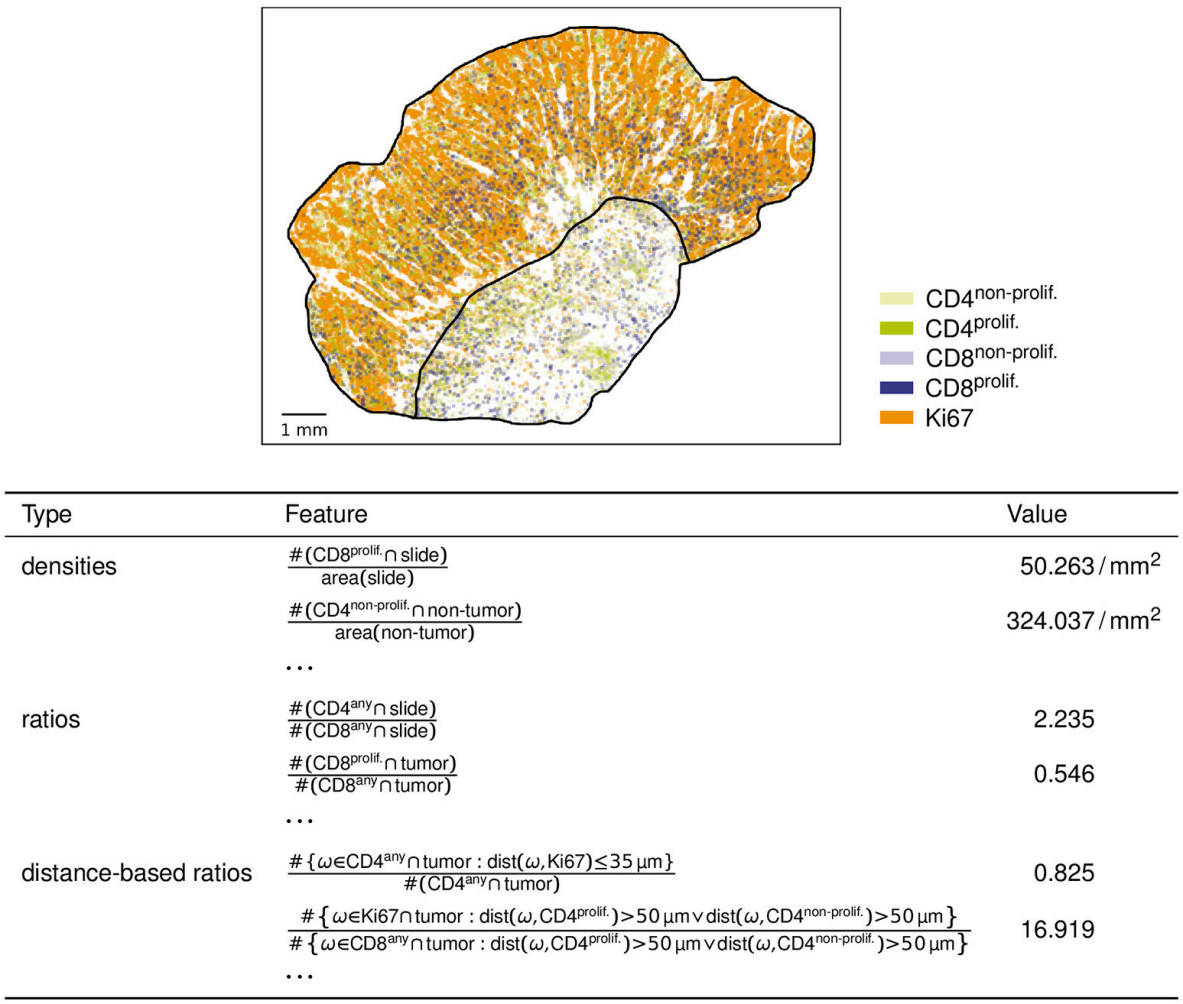
Example analysis results. For the slide with object data visualized above, the table shows six example features computed by our analysis.

As distance-based features involving distance criteria, we compute a number of ratios of marker counts involving distance criteria (of the form introduced above) in the numerator and (optionally) in the denominator. Two examples (cf. Figure 2) are

- the number of CD4 immune cells in the tumor compartment no farther than 35 μm from the closest Ki67 single-positive cell, divided by the total number of CD4 immune cells in the tumor compartment; and
- the number of Ki67 single-positive cells in the tumor compartment more than 50 μm from the closest (proliferating or non-proliferating) CD4 immune cell, divided by the number of (proliferating and non-proliferating) CD8 immune cells in the tumor compartment more than 50 μm from the closest (proliferating or non-proliferating) CD4 immune cell.

Following a data-driven approach, the combinations and distance thresholds considered here were chosen regardless whether any biological mechanisms are known that would motivate the specific choice.

Mathematically, such features in the most general form considered in this study can be expressed as

(9) $$\frac{\#\left\{\omega_{a}\in \Omega_{a}\cap R:\left\{\begin{array}{l} \vee_{\Omega \in M}\\ \wedge_{\Omega \in M} \end{array}\right\}\text{dist}\left(\omega_{a},\Omega \right){\{}_{>}^{\leq }\}\theta \right\}}{\#\left\{\omega_{b}\in \Omega_{b}\cap R:\left\{\begin{array}{l} \vee_{\Omega \in M}\\ \wedge_{\Omega \in M} \end{array}\right\}\text{dist}\left(\omega_{b},\Omega \right){\{}_{>}^{\leq }\}\theta \right\}},$$

where the choices of “or”/“and” and ≤ / > are independent of each other.

We used the 5,859 object combinations explained in the Supplementary Data Sheet 1, Section 1.2 and Table S1, combined with the distances from Equation (5) as upper and lower thresholds, respectively.

In principle, the reference objects M and the distance thresholds θ could, of course, be chosen independently for the denominator and the numerator as a further generalization of Equation (9). To keep the total number of distance-based features and the computational workload manageable, we use the slightly restricted form of Equation (9). This, however, is not a general limitation of our approach, merely of our current implementation, and has no biologically motivated reason. Note that distance-free object counts and global ratios introduced above can be viewed as a special case of a distance-based fraction using, e.g., an upper threshold for the distance larger than the specimen size or a lower threshold smaller than the minimal cell distance, so that all objects are included in the count.

#### 2.3.3. Implementation

Our feature analysis is implemented in Python in two main steps:

1. Data is imported, tissue compartments and the point objects contained therein are determined, and object-to-object distances are computed.
2. The features defined above are computed.

For the Boolean operations on polygons representing the tissue regions, we use the shapely library (21), which in turn uses the GEOS library (22). We also use shapely to determine the respective areas and the point-in-polygon relations representing whether an object lies in the tumor or the non-tumor tissue. For efficiently computing object-to-object distances, we use the KDTree data structure of SciPy (23). For storing the input data and the results obtained in the first analysis step, we use sqlite databases (24).

In the subsequent feature computation, we combine Python code and SQL database queries from within Python, e.g., for counting the number of objects with different distance thresholds. Due to the amount of data and the number of features, the feature analysis is computationally expensive. Profiling at the Python level helped us to find performance bottlenecks and optimize the implementation of the database interaction for speed. For this purpose, we mainly use two techniques: indexing of the database tables where appropriate, and caching of database query results: if no data gets written between repeated identical queries, the result can be assumed to be the same and can hence be cached. Write access can be prevented if only a single process at a time works with a given database. To further improve computational performance, we parallelize at the process level, i.e., we treat different slides separately in the feature analysis and write the results to separate databases that are merged only at the end.

#### 2.3.4. Example Values of Global Features

We obtain a total of 6,387 distance-based and density-based features (equipped with different distance thresholds), cf. Table 1 and the Supplementary Data Sheet 1, Table S1. There are strong redundancies between part of the different features considered, so any subsequent analysis should not view them as independent of one another. This is intended, so that biological effects can be pinpointed by single features rather than a combination of multiple orthogonal features.

**Table 1:** Number of features and percentages of different classes considered.

**Class**	**Density-based**	**Distance-based**	**Sum**
Global	528	5,859	6,387
Heterogeneity	8,448	93,744	102,192
Sum	8,976	99,603	108,579

*Generally, different values for distance and heterogeneity parameters lead to larger numbers of features in these classes*.

As example results, six of the feature values computed for one example slide are shown in Figure 2.

### 2.4. Heterogeneity Features

The computation of the features above only yields average values for the slide, tumor and non-tumor tissue; it does not capture heterogeneity across the tissue. Heterogeneity, however, might also contain valuable information that can be used to discriminate different types of cases. We thus define tilings of the tissue regions and compute different measures from descriptive statistics to quantitatively characterize heterogeneity of the spatial object distributions. We will refer to these features as “heterogeneity features” as opposed to the global features introduced above.

#### 2.4.1. Tile-Based Analysis

To characterize the heterogeneity of the global features introduced above at different length scales, we define square tiles covering the tissue regions with edge length 250, 500, and 1,000 μm. The tile sizes are chosen to be both large enough for the robust computation of features and small enough for the assessment of heterogeneity. In each tile for a fixed size, we first compute values of the base features. This is achieved by evaluating the formulas 1–4 and 6–9 for each tile as a region *R*. Subsequently, we compute descriptive statistical measures capturing different aspects of heterogeneity for the values of a fixed feature over the tiles of a fixed size, see section 2.4.2.

Distance thresholds in the global features do not respect tile boundaries. Respecting them would not be useful since biological cell-cell interactions happen in the tissue regardless of a tiling defined purely for assessment purposes. Hence, no additional computation of object-to-object distances is necessary.

**Figure 3:**
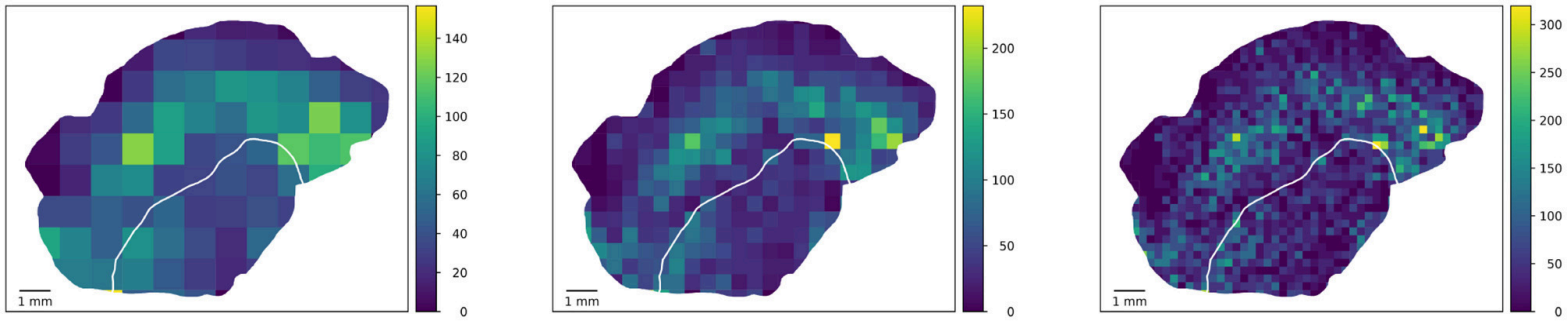
Square-based analysis. Computing a square-based feature (density of proliferating CD8 objects) in squares of different size captures clusters of different size. Note that the color maps are scaled to a maximum range for each image separately to enhance the visibility of variability. By using measures from descriptive statistics, we quantify heterogeneity independent of spatial patterns one might observe in the tile-based values.

Figure 3 shows the visibility of clusters (peaks of the density of proliferating CD8 objects as an example feature) at different square tile sizes. In order to rule out artifacts due to tiles with only small overlap with the tissue, only those tiles whose overlap with the tissue is at least 10 % of the tile area are considered in the statistical computations. An example for such an artifact can be seen in the left image in Figure 3 where the maximum is attained in a barely visible sliver at the top left where the tumor boundary hits the tissue boundary. This lower threshold of 10% overlap in the analysis is a rather arbitrary example choice and could be adapted. Also, it could be replaced by using the area overlap values as weighting factors in the statistical measures introduced below.

In addition to computing the statistical measures for all tiles, we restrict the evaluation to those tiles lying entirely inside the tumor part (i.e., not those overlapping with both tumor and non-tumor tissue). We hence capture the heterogeneity in the entire slide and only within the tumor, the non-tumor tissue alone was not considered in the tile-based evaluation. One consequence of this approach is that the tumor tissue is not represented fully and, in particular, that different parts of the tumor are considered at different length scales. This could be ameliorated by intersecting tiles with the tumor, however at the cost of more variable effective tile size for a given tile size.

This approach using tools from descriptive statistics intentionally drops the spatial information about the tiles, e.g., about whether “hot spots” of the feature are located in adjacent tiles or spread out through the tissue.

#### 2.4.2. Heterogeneity Measures

##### 2.4.2.1. Variation

As an immediate measure of the variation, we compute the coefficient of variation (COV; standard deviation divided by arithmetic mean) and the quartile coefficient of dispersion,

(10) $$\begin{array}{r} \text{QCD}=\frac{p_{75}-p_{25}}{p_{75}+p_{25}}, \end{array}$$

where *p*_*i*_ is the *i*th percentile.

The COV includes all data, but is sensitive to outliers and might be misleading as the underlying data is not necessarily normally distributed. In contrast, the QCD makes no assumptions about the distribution of values and is robust to outliers, but does not capture the data beyond the 25th and 75th percentile.

To reduce redundancy and make the heterogeneity values more comparable between different features, we compute the variation and percentiles relative to “typical values.” More precisely, we use the coefficient of variation (as described above) rather than the variance; and the QCD rather than the inter-quartile range (*p*_75_ − *p*_25_).

##### 2.4.2.2. Extrema

To quantify the relative values of extrema, i.e., the “height of peaks” and “depth of troughs,” we compute fixed percentiles (3, 5, 10, 90, 95, 97) divided by the median, resulting in relative percentiles RP 3, …, RP 97.

For each of the 2,129 global features per region (cf. Supplementary Data Sheet 1, Table S1), eight heterogeneity values in three different tile sizes are obtained, each of which is computed in the entire slide and restricted to the tumor, resulting in 2129 × 8 × 3 × 2 = 102, 192 heterogeneity features. Together with the global features in the slide, tumor, and non-tumor tissue, this yields a grand total of 108,579 features. An overview of the number of features considered in these classes is given in Table 1.

#### 2.4.3. Implementation

The tile-based feature computation is implemented as part of the two steps explained above, the computation of heterogeneities is a subsequent third step.

In addition to the tissue regions, tiles and object-in-tile relations are computed.
  We represent the square tiles as polygons and compute their intersection with the tissue again using the shapely library. The implementation from here onwards is generic in the sense that the tile polygons could have arbitrary shape and could overlap. Point-in-polygon (tile) relations are computed also using the shapely library. The tiles, their areas, and the object-in-tile relations are stored in the same sqlite database as introduced above.
Features are also computed per tile.
  Feature values per tile are computed in the same way as for the tissue regions, but now with counts restricted to the respective tiles, and also stored in the database. One difference is that this analysis frequently produces non-finite values due to division by zero (no objects of specific type in a tile, in particular for less frequent objects and small tiles). We handle this transparently using standard IEEE floating point arithmetics (via numpy in Python), resulting in *x*/0 = ∞, *x*≠0 and 0/0 = NaN (not a number) and suitable conversion when storing/retrieving data from the sqlite databases.
Heterogeneities are computed.
  This is based on querying the database for the per-tile values of each global feature and for each tile size. We filtered out the non-finite values before computing statistical quantities. The different heterogeneity quantities are also stored in the database.

### 2.5. Identification of Discriminatory Features

After computing 108,579 features for a dataset, we rank all features according to their discriminatory power. This takes into account two effects: how well can a single threshold separate the feature values of the two classes, MSI and MSS, and to what extent can the feature be computed for the cases in the first place. More generally, this also enables the identification of predictive biomarkers by comparing features between responders and non-responders, or the identification of markers capturing treatment effects, by comparing features pre-treatment and post-treatment, given suitable data.

#### 2.5.1. Quantification of Discriminatory Power

For a simple decision rule whether a case should be considered as MSI or MSS based on a single feature, one could apply a decision tree classifier of depth 1, also denoted decision stump (25). This algorithm would determine a single threshold upon which the two classes are separated best. However, there is no unique criterion for “good” separation of the classes, all conceivable criteria require a trade-off between specificity and sensitivity, i.e., a compromise to what extent MSI cases mis-classified as MSS can be tolerated and vice versa. A useful compromise depends on the specific biological question and the potential imbalance (different number of cases for the classes) at hand.

A standard approach to quantify the well-posedness of binary classification tasks (i.e., how good such compromises can be at all) is to compute the area under the curve of the receiver operating characteristic curve (ROC-AUC) (26). The ROC-AUC is a number between 0 and 1, where 0.5 is the worst case indicating that no separation is possible, and the extreme values 0 and 1 are the best cases indicating that all feature values for one class are smaller or larger than all the feature values for the other class. As these two cases are equivalent in terms of separation performance, we replace ROC-AUC by 1 minus ROC-AUC if ROC-AUC < 0.5.

#### 2.5.2. Quantification of Overall Performance

Features that can only be computed on a subset of the available data (e.g., due to the lack of a non-tumor region in the slide or a zero object count appearing in the denominator of a ratio feature) are less useful than features that can always be computed.

To obtain a more useful overall performance measure (OPM) taking into account the availability of features on the set of slides, we define an overall performance measure as follows: We scale the ROC-AUC to a range between 0 (worst performance) and 1 (best performance), scaled by the availability of the features in the two classes.

Mathematically, this OPM is defined as

(11) $$\begin{array}{r} \text{OPM}=2\cdot (\left(\text{ROC-AUC}\right)-0.5)\cdot \frac{C_{1,f}}{C_{1}}\cdot \frac{C_{2,f}}{C_{2}}, \end{array}$$

where *R* is the ROC-AUC, *C*_1_ and *C*_2_ are the total counts of available slides of classes 1 and 2, respectively, and *C*_1, *f*_ resp. *C*_2, *f*_ are the counts of the respective slides for which feature *f* is available. This formula scales the ROC-AUC to a range between 0 and 1 (mapping the “worst” value for *R*, 0.5, to a performance measure of 0), and scales the performance by the fractions of slides for which the feature is available in the classes of interest. Separating these two fractions rather than using an overall feature computability fraction is capable of reflecting imbalanced class sizes.

#### 2.5.3. Implementation

This evaluation is implemented using the ROC-AUC computation of scikit-learn (27, 28) in Python as the basis for our overall performance measure. We obtain the individual feature values from the database containing the feature results, and store the ROC-AUC values in the database for all features. We compute OPM values for each of the 108,579 features obtained in the analysis above in a postprocessing step, and rank the features according to these OPM values.

## 3. Results

As a verification, we first applied our analysis approach to a targeted synthetic dataset designed such that a given feature is known to be perfectly discriminatory (section 3.1). We then applied the analysis to the MSI/MSS dataset described in section 2.1, reporting five features of high discriminatory power (section 3.2), and giving an overview on the performance of different types of features (section 3.3). Finally, we estimated the significance of these results, i.e., whether this discriminatory power was really due to structure in the data and not merely a consequence of computing a large number of features (section 3.4).

### 3.1. Verification of the Approach

To verify the overall approach, we picked one specific feature, generated synthetic slides differing in this specific feature, and checked that the assessment of discriminatory power found this feature to be highly discriminatory. This analysis also demonstrates that the data-driven approach is capable of discovering discriminatory features that are hard to discern or assess visually.

As synthetic slides without any intended biological meaning, we used simple square tissue sections of 1 mm edge length without tumor compartment with generally realistic object densities for exactly two object types (1,000 Ki67 objects, 50 proliferating CD8 objects). To construct slides in a detectably different way, we uniformly distributed objects within the tissue, subject to two different distance constraints: In group A of synthetic slides, a distance >15 μm between Ki67 and all 50 CD8 objects was enforced. In group B, this criterion was used for 45 CD8 objects, 5 of the CD8 objects were positioned within 15 μm of a Ki67 object. Additionally, a minimum distance of 10 μm between all object points was enforced in both groups.

**Figure 4:**
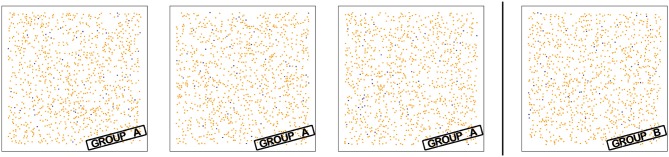
Slides for verification experiment. The synthetic slides using 1 mm^2^ tissue in two groups are constructed such that the fraction of Ki67 objects (orange) within 15 μm of the closest proliferating CD8 object (dark violet) differs the two groups: This fraction is 0 in group A and ≥0.01 in group B. The digital approach successfully discriminates the visually similar slides.

This led to two groups of slides that are not easily distinguished by visual assessment (cf. Figure 4), but where the fraction of Ki67 objects within 15 μm of the closest proliferating CD8 object clearly discriminates the two groups: by construction, this feature is exactly 0 in group A and at least 5/50 = 0.01 in group B.

In fact, the analysis of discriminatory power identified this feature as perfectly discriminatory with an OPM of 1.0 (ROC-AUC 1.0, feature available on all slides). There were no other object types (e.g., CD4) present in the synthetic slides, but the full set of features was computed. Hence, multiple features were equivalent (e.g., CD8^any^ = CD8^prolif.^ in these synthetic slides) and thus equivalently discriminatory.

### 3.2. Discriminatory Features for the MSI/MSS Dataset

**Figure 5:**
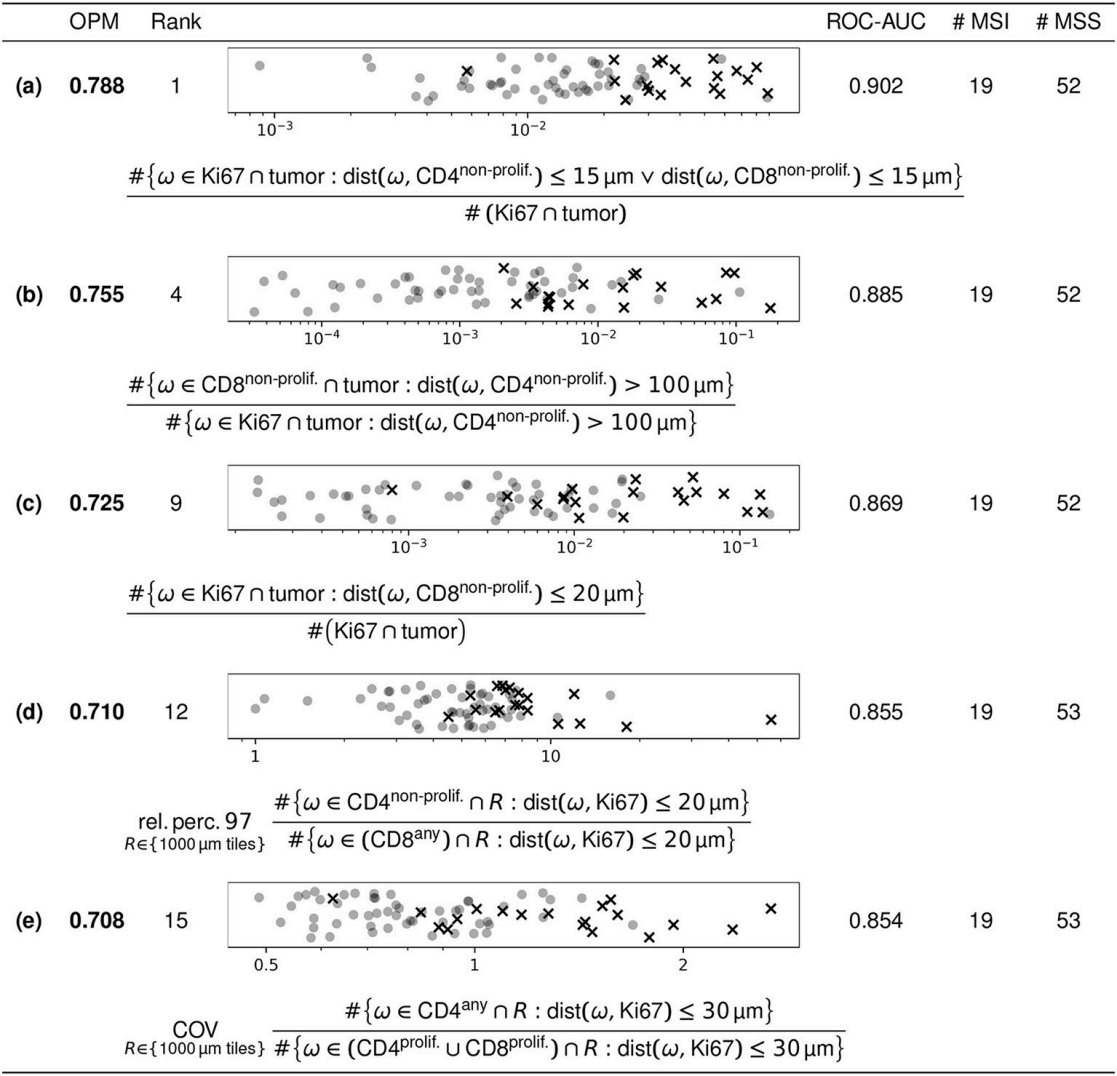
Selected features with high discriminatory power. Of the 108,579 features included in our analysis, the figure shows five selected features of high discriminatory power. The parameters for the features shown are explained in the text. The scatter plots show the feature values of the respective feature (all of which are dimensionless), with MSI and MSS as crosses and circles, respectively. Data points are uniformly randomly scattered in vertical direction and plotted on a logarithmic horizontal scale.

For our case study using the MSI/MSS dataset described in section 2.1, five features with high discriminatory power in terms of OPM are described below. Figure 5 lists the quantitative results of this assessment and shows scatter plots visualizing the feature values, as well as a mathematical description of the features as formulas:

(a) Fraction of Ki67 single-positive markers in the tumor at most 15 μm from closest non-proliferating either CD4 or CD8 marker (i.e., ratio of the number of these over the number of all Ki67 single-positive markers in the tumor).
(b) Ratio of the number of non-proliferating CD8 markers in the tumor at least 100 μm from the closest non-proliferating CD4 marker over the number of Ki67 single-positive markers in the tumor subject to the same distance criterion (at least 100 μm from the closest non-proliferating CD4 marker).
(c) Fraction of Ki67 single-positive markers in the tumor at most 20 μm from closest non-proliferating CD8 marker (i.e., ratio of the number of these over the number of all Ki67 single-positive markers in the tumor).
(d) The 97th relative percentile of the following ratio in squares of 1,000 μm edge length across the entire slide: the number of non-proliferating CD4 markers at most 20 μm from the closest Ki67 single-positive marker over the number of (proliferating or non-proliferating) CD8 markers subject to the same distance criterion (at most 20 μm from the closest Ki67 single-positive marker).
(e) The coefficient of variation of the following ratio in squares of 1000 μm edge length across the entire slide: the number of (proliferating or non-proliferating) CD4 markers at most 30 μm from the closest Ki67 single-positive marker over the number of proliferating T cell (CD4 or CD8) markers subject to the same distance criterion (at most 30 μm from the closest Ki67 single-positive marker).

These five features were selected from the 20 features with largest OPM values (listed in the Supplementary Data Sheet 1, Figures S1–S3) by omitting a number of features conceptually similar to feature (a). The five features selected here were available for almost all cases. One of the MSS cases did not have a tumor compartment annotated, so the features (a–c) could be computed for all except one MSS case.

### 3.3. Overall Feature Performance

To compare the general discriminatory power of features in different classes (cf. Table 1), we report the numbers of potentially predictive features in the respective classes in Table 2.

**Table 2:** Numbers of potentially predictive features in the different classes of features.

**Class**	**Density-based**	**Distance-based**
Global	0	157
Heterogeneity	35	323

*The total numbers of features per class are listed in Table 1*.

In order to further investigate the influence of the threshold in cell-to-cell distances, we compared how many potentially predictive features we obtained depending on the distance thresholds and for the density-based features. For this purpose, we considered a threshold of OPM≥0.6. In case of features available on all slides, this corresponds to ROC-AUC≥0.8, an even higher ROC-AUC is required for features not available on all slides. With this criterion, 515 of the 108,579 biomarkers in total were found to be potentially predictive. The results in Table 3 indicate that there were few potentially predictive density-based features and that more potentially predictive features for very small distance threshold were obtained.

**Table 3:** Numbers of potentially predictive features based on cell-to-cell distances with different distance thresholds and density-based features.

Distance threshold θ in μm	15	20	25	30	35	50	100	(none)
# Potentially predictive features	94	69	62	55	70	67	63	35

*The total numbers of features included in our analysis were 14,229 for each of the distance thresholds and 8,976 density-based features*.

### 3.4. Estimated Significance of the Discriminatory Power

We further assessed our feature selection method and the results for the MSI/MSS use case to show that the observed discriminatory power is meaningful and not merely a random finding in our large number of features. For this purpose, we followed a validation strategy similar to the ones suggested in Horvatovich and Bischoff (29), Guyon and Elisseeff (30), using pseudo-randomly generated data of the same size as the real feature data. Our ROC-AUC-based assessment is invariant under monotonic transformations of the feature values, so we chose a uniform distribution for the random data. It had the same assignment to classes (MSI/MSS) as the real data, was fed to the feature selection method, and finally the respective OPM values were computed.

**Figure 6:**
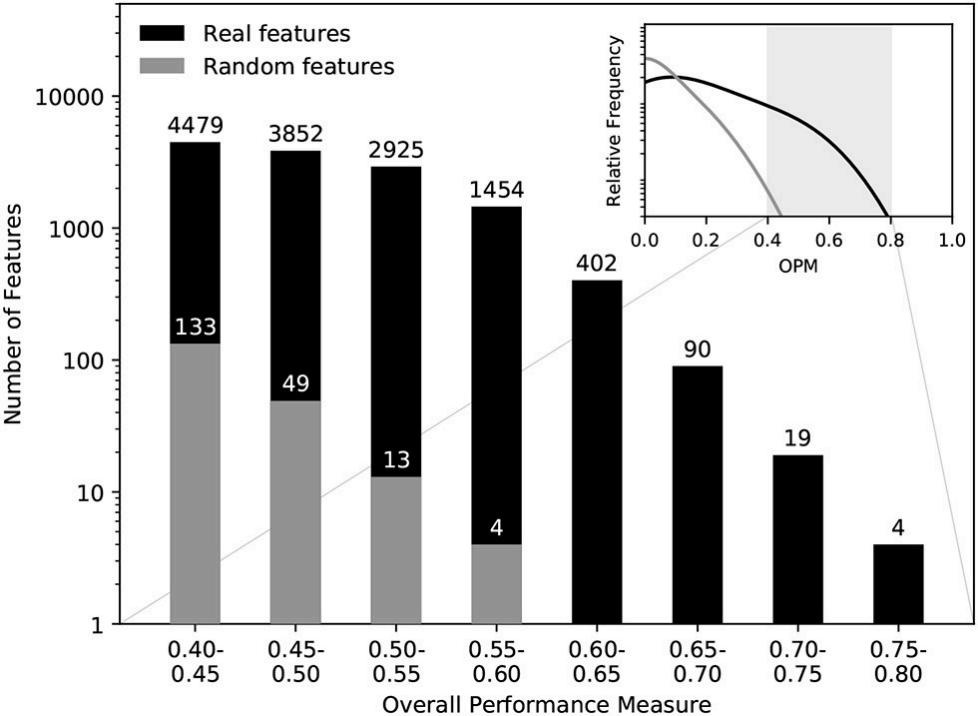
Significance assessment of the discriminatory power. Comparing the OPM values obtained for the MSI/MSS data to OPM values obtained for comparable random feature values, we can see that features with OPM≥0.6 are virtually guaranteed to be true positives in terms of discriminatory power.

Figure 6 shows the distributions of OPM values for the real data and the randomly generated data. The histogram of the randomly generated data does not include any possible biomarkers and therefore gives a good indication for the number of false positives to expect in the real data (29). Comparing the histograms, we can conclude that features with OPM≥0.6 virtually eliminated the chance of false positives.

## 4. Discussion

### 4.1. Data-Driven Feature Discovery

The results in section 3.1 show that the proposed approach can identify cell interactions of predictive relevance that are hard to find visually (cf. Figure 4). Thus, our data-driven approach can serve as a useful tool complementary to visual exploration and knowledge-based biomarker discovery.

#### 4.1.1. Results for the MSI/MSS Dataset

The results obtained for the MSI/MSS dataset in section 3.2 reveal general insights about immune contexture features. Very different features can exhibit high discriminatory power (cf. Figure 5).

Both features (a) and (c) reflect differences between MSI and MSS cases in term of infiltrating T cells among proliferating tumor cells. Biologically, both CD8 and CD4 T cells are expected to be highly discriminatory since co-localization of T cells and tumor cells reflects the presentation of tumor antigens, which is presumably higher in MSI cases, and the subsequent response of the immune system.

Feature (b) potentially reflects the role of CD4 T cells in modulating the function of CD8 T cells, assuming that distances above 100 μm are sufficiently far to prevent said modulation. One could interpret it in a way that CD4 T cells that are close to CD8 T cells modulate their function which in turn influences CD8 engagement with proliferating tumor cells cf. (31).

Features (d) and (e) are heterogeneity features and thus less intuitive to interpret. In a nutshell, feature (d) quantifies peaks (at the millimeter length scale) of the ratio of the number of non-proliferating CD4 T cells ajdacent to tumor cells over the number of T effector cells adjacent to tumor cells. Feature (e) quantifies the variation (also at the millimeter length scale) of the ratio of the number of CD4 T cells in the vicinity of tumor cells over the number of proliferating T cells in the vicinity of tumor cells.

The high discriminatory power of features (b, d, and e) corroborates the importance of CD4 cells, whose interactions have been investigated (32) but not fully understood yet. Further investigations are needed to elucidate the role of heterogeneity of the cell populations involved in features (d) and (e).

#### 4.1.2. Assessment of Different Feature Classes

##### 4.1.2.1. Density-based vs. distance-based features

All features with OPM>0.7 (including the five shown in Figure 5) are based on cell-to-cell distances. Tables 2, 3 corroborate that distance-based features generally have higher discriminatory power than density-based features. This confirms previous findings (7, 8) that cell-to-cell distances may contain essential predictive information.

##### 4.1.2.2. Distance thresholds

The distance threshold occurring in the five features shown in Figure 5 are generally small, ranging down to the minimum value of 15 μm we used in the feature analysis. The typical cell size is a lower bound for meaningful thresholds in this context, so no benefit can be expected from even smaller thresholds. This observation is corroborated by the numbers in Table 3, showing that more potentially predictive features are found for distance thresholds of 15 μm than for other thresholds. However, these cumulative numbers do not differentiate between different cell types. These findings suggest that direct interactions between neighboring cells are most relevant for the distinction of MSI and MSS cases.

##### 4.1.2.3. Global and heterogeneity features

The highly discriminative features shown in Figure 5 comprise both global and heterogeneity features. This trend is corroborated by the numbers in Table 2, indicating that measuring feature heterogeneity is useful for assessing the tumor microenvironment.

##### 4.1.2.4. Tissue compartments

The OPM does not only take the predictive power of features into account, but also the number of cases for which a feature can be computed. Since only 16 of the 19 MSI and 42 of the 53 MSS slides contain any non-tumor tissue regions, features derived from the non-tumor compartment generally had lower OPM values.

Non-tumor regions being available only in part of the dataset is not an inherent limitation of these features, but a limitation of the evaluation data. The most predictive feature derived from the non-tumor compartment is the fraction of Ki67 single-positive markers at most 15 μm from the closest non-proliferating either CD4 or CD8 marker, i.e., the same feature as in (a) but evaluated in the non-tumor compartment. This feature has ROC-AUC = 0.890, which is comparable to the ROC-AUC values of the highly discriminative features derived from the tumor compartment or the entire tissue listed in Figure 5. This confirms previous findings (33–35) that not only inflammation in the tumor itself but also immune infiltration in its surrounding stroma has prognostic and predictive value.

##### 4.1.2.5. Feature values

In Figure 5, one can observe that the feature values of the MSI cases are generally larger than the feature values of the MSS cases (the crosses tend to the right of the threshold, the dots to the left). This is not a meaningful trend and does not generalize beyond the five features shown, it is merely a consequence of the terms in the numerator and denominator in the fractions describing the respective feature.

### 4.2. Limitations and Outlook

Our findings of potentially predictive features for the MSI/MSS dataset (section 3.2) should be interpreted and generalized with care. On the one hand, only a single, small cohort was used for the evaluation. On the other hand, the predictive power was not assessed toward actual therapy response but toward MSI as a surrogate target variable.

The large number of features included in the analysis, and the numbers of features in the respective classes (cf. Table 1) should be compared with care. By design, our data-driven approach included a large number of features because they could be computed from the input data and not because they were expected to be predictive based on prior knowledge.

The findings about feature classes (sections 3.3 and 4.1.2) should also be interpreted with care, again due to the limited scope of the evaluation. We can conclude that considering spatial context, i.e., cell-to-cell distances and feature heterogeneity, yielded potentially predictive biomarkers for our dataset. Thus, such features may be useful for other datasets as well.

#### 4.2.1. Feature Analysis

In this study, cells of a limited number of types are considered. Further cell types and features are conceivable, reflecting more general relations between different object types. Moreover, the cell objects are represented by point objects. This could be extended by including cell morphological features, taking into account the key information a pathologist would use for visual assessment of the tissue. However, this requires a reliable segmentation of cell shapes which is generally very challenging.

Our approach to quantify heterogeneity uses tools from descriptive statistics. Hence, spatial patterns with the same distribution of feature values cannot be differentiated by this approach. This could be tackled by texture features that characterize the spatial arrangement of tile values in a quantitative manner (10).

Further features could potentially be derived by comparing the same heterogeneity measure for the same base feature for more different tile sizes, which could help identify “characteristic length scales” in the object data.

**Figure 7:**
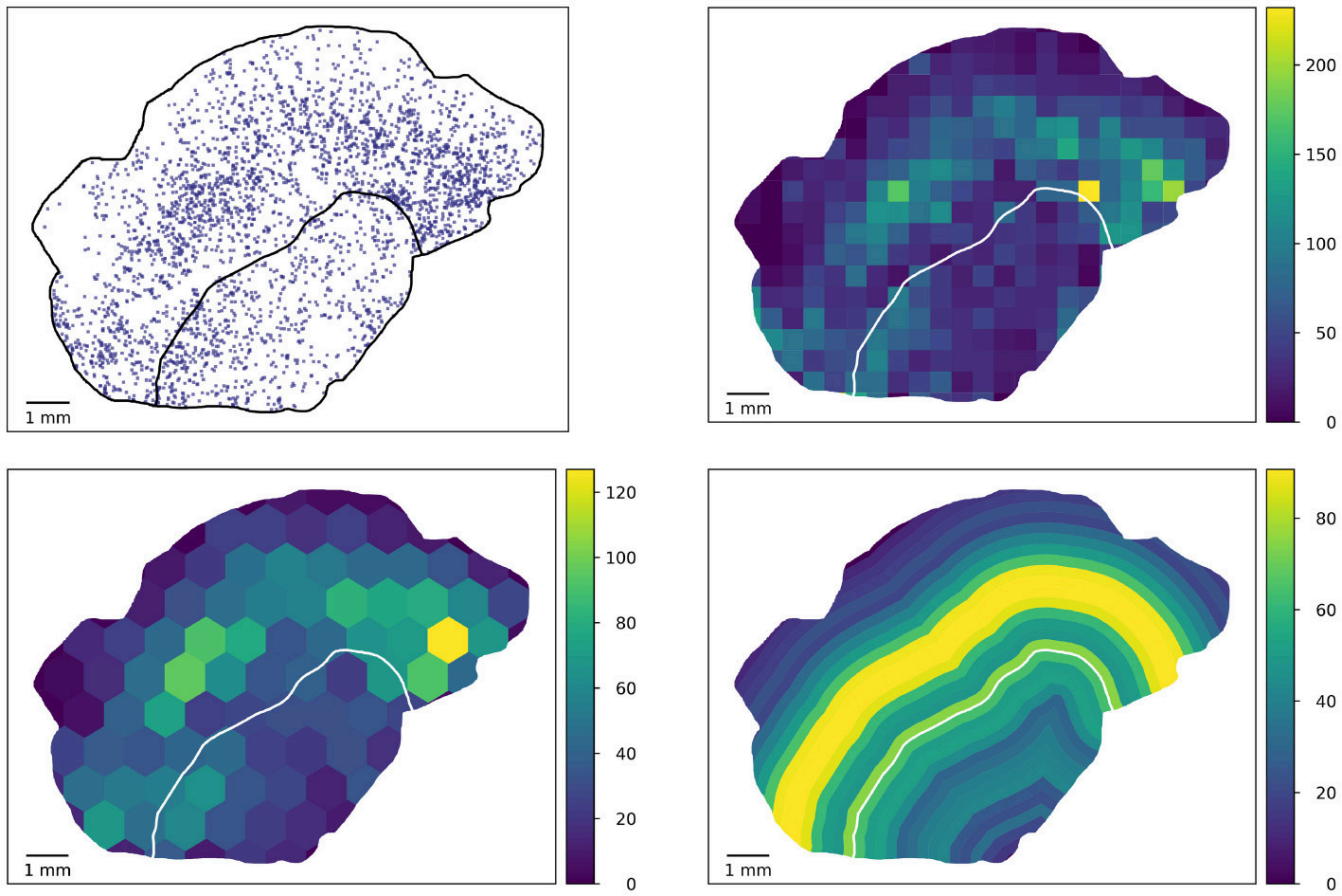
Potential extension of the tile-based analysis. Densities of proliferating CD8 objects (top left) can be computed in generic tiles, e.g., geometrically defined squares (top right) used in this study, hexagons (bottom left, mock-up), or biologically motivated equidistant rings at the tumor boundary (bottom right).

The tiling approach in the heterogeneity characterization is generic in the sense that arbitrary polygons can be used, e.g., hexagons (11), bands near a tumor boundary (if any), or approximations of circles. Some possibilities are illustrated in Figure 7, but also overlapping tilings are conceivable (albeit not straightforward to visualize).

The ring-based analysis indicated in Figure 7 gives rise to the possibility of considering further features with distances to the tumor boundary, or an invasive margin which could also be annotated in the slides and considered in the feature analysis. This would be of high biological interest. However, it requires a robust annotation of said invasive margin and moreover has the difficulty that distances to tumor boundaries can only be computed for the tumor and non-tumor tissue present in the specimen at hand which typically is a 2D section through part of the tumor.

#### 4.2.2. Assessment of Discriminatory Power

The prognostic or predictive power of a biomarker critically depends on the classification method. To enable the formulation of useful clinical decision rules, the assessment of discriminatory power was limited to single features and cutoff-based decision rules. It would be straightforward to assess the predictive power of feature combinations or more complex classifiers. Such a more complex approach may be useful in cases where single features are not prognostic, but bivariate classification allows patient stratification (36). However, more complex approaches make it more difficult to translate results into clinical practice. Also, an increased number of feature combinations and more complex classifiers greatly increase the risk of random findings, thus making a significance assessment even more important.

For more complicated classifiers, cross-validation techniques would have to be employed to mitigate the risk of overfitting for more complex classifiers (30). Moreover, the approach for estimating significance of the discriminatory power would have to be adapted. In this case, the significance assessment described in section 3.4 would have to take into account the distribution of the actual feature values, estimated based on the observed feature values oblivious to the classes.

Furthermore, our OPM to quantify the predictive power is just one possibility; feature availability could be considered differently to assess overall performance. Our criterion of OPM≥0.6 used in the assessment in section 3.3 was confirmed to be useful in Figure 6, but is still a somewhat arbitrary choice that may need to be changed for other applications.

#### 4.2.3. Robustness

Only robustly computable features are useful for classification of future, yet unknown, data. A number of aspects needs to be taken into account in this context. Regarding the input to the feature discovery, histological specimens are only incomplete samples of the diseased organs; staining and imaging in different labs may introduce variation in the image data; and the annotation of tissue regions and detection of cell objects and their types may be imperfect. The latter is particularly relevant for rare cell types. Also within the feature discovery process, parameters of the analysis may have an impact on the feature results: distance thresholds; shape, orientation, and position of the tiling relative to the tissue specimen; percentiles; but also parameters of the cross-validation.

Using estimates of the variability of the data in the respective aspects, one could assess the robustness by suitable Monte Carlo simulations. A thorough robustness investigation, however, should also include the interplay of different sources of variability and goes beyond the scope of the present study.

#### 4.2.4. Application for Biomarker Discovery

The biomarker discovery approach presented in this study is used for an example application using histologies of a specific cancer type (human colorectal cancer), with specific cell types stained and detected (Ki67, CD4, CD8), considering specific binary end points (MSI, MSS), in one specific patient cohort. The approach is oblivious to all these aspects and can be applied for other cancer types, cell types, end points, and cohorts–provided that suitable data is available.

Besides the full discovery approach, also the individual building blocks can be applied in biomarker research. Precisely describing features derived from cell markers is also applicable for hypothesis-driven research based on hand-designed features. Quantitatively assessing discriminatory power is generally useful in case of incompletely available feature data and imbalanced class sizes. Carefully assessing the significance of discriminatory power is beneficial in exploratory, data-driven research where large numbers of potential biomarkers are considered.

## 5. Conclusion

The data-driven approach simplifies the identification of promising IC biomarker candidates from histological datasets with immune cells annotations, tumor compartment annotations, and clinically relevant endpoints. It works without having a specific hypothesis about the prognostic or predictive value of an immunological process and does not require defining features to capture this process. The approach is agnostic toward tumor biology and can identify cell interactions of predictive relevance that are not straightforward to find by visual inspection.

In an evaluation on a cohort of colorectal cancer patients, the identified promising biomarker candidates include features based on cell-to-cell distances and spatial heterogeneity. This finding corroborates earlier findings that spatial tissue context is essential for predicting therapy response. In fact, most methods used to describe the tumor immune status today rely on quantity and ignore the spatial relationship. This underlines the value of histology complementary to diagnostic methods without spatial resolution, such as genetic sequencing.

It appears unlikely that all identified biomarkers are false positives, even though they are identified in a data-driven manner using a small dataset. Nevertheless, biological interpretation and further prospective studies are necessary to confirm their validity. The most promising biomarker candidates in our example cohort comprise quantitative descriptions of co-localizations between CD8 and tumor cells (confirming previously known relevance) and the impact of CD4 cells on the anti-tumor immune response (pointing in a promising new research direction).

Most biomarker studies share the same challenges, including finding the right candidate features and feature selection strategy. Researchers can therefore take guidance from the described approach to accelerate their biomarker research.

## Data Availability Statement

All datasets used in this study are included in the Supplementary Material.

## Author Contributions

EA, FG, KK, and OG initiated the project. EA, KK, and OG conceived the idea of data-driven biomarker approach and provided direction, guidance and provided data. EA and KK annotated slides and verified data quality. LS developed and implemented the feature analysis. AH and NW developed and implemented the assessment of predictive power. EA and KK provided biological interpretation. AH, EA, KK, LS, and NW wrote the manuscript. FG, HH, OG, and SH revised the manuscript. All authors read and approved the submitted version.

### Conflict of Interest Statement

The authors declare that the research was conducted in the absence of any commercial or financial relationships that could be construed as a potential conflict of interest.

## Supplementary Material

Data Sheet 1.PDF Detailed lists of cell types considered in the MSI/MSS dataset and a more detailed presentation of potentially predictive features for the dataset.

Data Sheet 2.zip The synthetic dataset used for verifying the approach (section 3.1; Figure 4) is provided as a collection of text files describing regions, markers, and metadata.

Data Sheet 3.zip The MSI/MSS dataset used for the identification and assessment of discriminatory features (sections 3.2, 3.3, and 3.4) is provided as a collection of text files describing regions, markers, and metadata (part 1/3).

Data Sheet 4.zip MSI/MSS dataset, part 2/3.

Data Sheet 5.zip MSI/MSS dataset, part 3/3.
