# Supplementary material for: Data-Driven Discovery of Immune Contexture Biomarkers
Source: Front Oncol. 2018 Dec 18;8:627. doi: 10.3389/fonc.2018.00627 (PMC6305402; doi:10.3389/fonc.2018.00627)
Supplement: Data Sheet 1.PDF — Detailed lists of cell types considered in the MSI/MSS dataset and a more detailed presentation of potentially predictive features for the dataset. [file Data_Sheet_1.PDF]

Supplementary Material: Data-Driven Discovery of Immune Contexture Biomarkers

1 DETAILS ON BASE FEATURES

1.1 Density-Based Features

Counts and Densities

Object counts and densities are computed for all individual objects

- Ki67: Ki67-single-positive objects (proliferating cells, used as a surrogate for proliferating tumor cells)
- $\mathrm{CD4}^{\mathrm{prolif.}}$: CD4-positive, Ki67 positive objects (proliferating T helper cells)
- $\mathrm{CD4}^{\mathrm{non\text{-}prolif.}}$: CD4-positive, Ki67-negative objects (T helper cells)
- $\mathrm{CD8}^{\mathrm{prolif.}}$: CD8-positive, Ki67-positive objects (proliferating T cells)
- $\mathrm{CD8}^{\mathrm{non\text{-}prolif.}}$: CD8-positive, Ki67-negative objects (T cells)

as well as the object combinations

- $\mathrm{CD4}^{\mathrm{any}} = \mathrm{CD4}^{\mathrm{prolif.}} \cup \mathrm{CD4}^{\mathrm{non\text{-}prolif.}}$
- $\mathrm{CD8}^{\mathrm{any}} = \mathrm{CD8}^{\mathrm{prolif.}} \cup \mathrm{CD8}^{\mathrm{non\text{-}prolif.}}$
- $\mathrm{CD4}^{\mathrm{prolif.}} \cup \mathrm{CD8}^{\mathrm{prolif.}}$
- $\mathrm{CD4}^{\mathrm{non\text{-}prolif.}} \cup \mathrm{CD8}^{\mathrm{non\text{-}prolif.}}$
- $\mathrm{CD4}^{\mathrm{any}} \cup \mathrm{CD8}^{\mathrm{any}}$,

resulting in a total of 10 count and 10 density features in Equation 1 and Equation 2 in the main article, respectively, per tissue region.

Global Ratios

As global ratios in Equation 3 in the main article, we use the following object combinations (Ω_a, Ω_b) with $\Omega_a \neq \Omega_b$

- Ki67
- $\mathrm{CD4}^{\mathrm{prolif.}}$
- $\mathrm{CD4}^{\mathrm{non\text{-}prolif.}}$
- $\mathrm{CD8}^{\mathrm{prolif.}}$
- $\mathrm{CD8}^{\mathrm{non\text{-}prolif.}}$
- $\mathrm{CD4}^{\mathrm{any}}$
- $\mathrm{CD8}^{\mathrm{any}}$
- $\mathrm{CD4}^{\mathrm{prolif.}} \cup \mathrm{CD8}^{\mathrm{prolif.}}$
- $\mathrm{CD4}^{\mathrm{non\text{-}prolif.}} \cup \mathrm{CD8}^{\mathrm{non\text{-}prolif.}}$
- $\mathrm{CD4}^{\mathrm{any}} \cup \mathrm{CD8}^{\mathrm{any}}$
- $\mathrm{CD4}^{\mathrm{prolif.}} \cup \mathrm{Ki67}$

Feature Type			# Features per R and θ	# Features per R	Total # Features
Density-Based	Counts			10	30
	Densities			10	30
	Ratios			156	468
	Total			176	528
Distance-Based	of the form in	Eq. S1	13	91	273
		Eq. S2	144	1008	3024
		Eq. S3	38	266	798
		Eq. S4	52	364	1092
		Eq. S5	32	224	672
	Total		279	1953	5859
Total				2129	6387

Table S1. Numbers of features for the different types of features. Density-based features can be evaluated in each of three regions R. Distance-based features are additionally parameterized by the distance threshold θ, where we consider seven different thresholds.

- $\text{CD8}^{\text{prolif.}} \cup \text{Ki67}$
- $\text{CD4}^{\text{prolif.}} \cup \text{CD8}^{\text{prolif.}} \cup \text{Ki67}$,

resulting in 13 (possibilities for Ω_a) $\times$ 12 (possibilities for $\Omega_b \neq \Omega_a$) $=$ 156 object combinations, again per tissue region.

In total, we thus obtain 176 features per tissue region, see Table S1.

1.2 Distance-Based Features

The distance-based features considered in our analysis can be grouped in the following five types. In the formulas below, R refers to the tissue regions described in Section 2.2 and θ denotes one of the thresholds introduced in Equation 5 in the main article.

1. Distance-based object fractions of the form

$$\frac{\#\{\omega_a \in \Omega_a \cap R : \text{dist}(\omega_a, \Omega_b) \leq \theta\}}{\#\{\text{all } \omega_a \in \Omega_a \cap R\}}, \tag{S1}$$

   where only the ‘within’ relation (i.e., $\leq$) is used as the ‘above’ relation (i.e., $>$) is merely the complement (1 minus the fraction for ‘within’) and does not contain additional information.

   Here, we used the combinations (Ω_a, Ω_b) listed in Table S2 which results in 13 features per distance threshold and for each tissue region.

2. Ratios of two distance-based object counts of the form

$$\frac{\#\left\{\omega_a \in \Omega_a \cap R : \text{dist}(\omega_a, \text{Ki67}) \left\{\begin{matrix}\leq \\ >\end{matrix}\right\} \theta\right\}}{\#\left\{\omega_b \in \Omega_b \cap R : \text{dist}(\omega_b, \text{Ki67}) \left\{\begin{matrix}\leq \\ >\end{matrix}\right\} \theta\right\}} \tag{S2}$$

   with the same distance criterion (same inequality, same threshold, same ‘second’ object type) in the numerator and denominator.

   Here, we used the combinations (Ω_a, Ω_b) with $\Omega_a \neq \Omega_b$ in

- $\mathrm{CD4}^{\mathrm{any}}$
- $\mathrm{CD4}^{\mathrm{prolif.}}$
- $\mathrm{CD4}^{\mathrm{non\text{-}prolif.}}$
- $\mathrm{CD8}^{\mathrm{any}}$
- $\mathrm{CD8}^{\mathrm{prolif.}}$
- $\mathrm{CD8}^{\mathrm{non\text{-}prolif.}}$
- $\mathrm{CD4}^{\mathrm{any}} \cup \mathrm{CD8}^{\mathrm{any}}$
- $\mathrm{CD4}^{\mathrm{prolif.}} \cup \mathrm{CD8}^{\mathrm{prolif.}}$
- $\mathrm{CD4}^{\mathrm{non\text{-}prolif.}} \cup \mathrm{CD8}^{\mathrm{non\text{-}prolif.}}$

which results in 2 (inequalities) $\times$ 9 (possibilities for Ω_a) $\times$ 8 (possibilities for $\Omega_b \neq \Omega_a$) $=$ 144 features per distance threshold and for each tissue region.

3. Fractions where the 'second' object type is a combination of objects of the form

$$\frac{\#\left\{\omega_a \in \Omega_a \cap R : \left\{\begin{smallmatrix}\bigvee_{\Omega\in\mathcal{M}} \\ \bigwedge_{\Omega\in\mathcal{M}}\end{smallmatrix}\right\} \operatorname{dist}(\omega_a, \Omega) \leq \theta\right\}}{\#\{\text{all } \omega_a \in \Omega_a \cap R\}}, \tag{S3}$$

where again the 'above' relation would just be the complement and was thus omitted.

Here, we used the combinations $(\Omega_a, \mathcal{M})$ listed in Table S3. This results in 2 ('or'/'and') $\times 19 = 38$ features per distance threshold and for each tissue region.

4. Object ratios with a distance criterion only in the numerator and different object types in the denominator of the form (hence not denoted 'fraction')

$$\frac{\#\left\{\omega_a \in \Omega_a \cap R : \left\{\begin{smallmatrix}\bigvee_{\Omega\in\mathcal{M}} \\ \bigwedge_{\Omega\in\mathcal{M}}\end{smallmatrix}\right\} \operatorname{dist}(\omega_a, \Omega) \left\{\begin{smallmatrix}\leq \\ >\end{smallmatrix}\right\} \theta\right\}}{\#\{\text{all } \omega_b \in \Omega_b \cap R\}}. \tag{S4}$$

We use the combinations $(\Omega_a, \mathcal{M}, \Omega_b)$ listed in Table S4, where the distinction 'or'/'and' only applies for object set combinations $\mathcal{M}$ consisting of more than one object type, here denoted as 'combined $\mathcal{M}$'. This results in 2 (inequalities) $\times$ $\big[$2 ('or'/'and') $\times$ 4 (for combined $\mathcal{M}$) $+$ 18 (for non-combined $\mathcal{M}$)$\big] = 52$ features per distance threshold and for each tissue region.

5. Object ratios with the same distance criterion in the numerator and the denominator as already described in Equation 9 in the main article,

$$\frac{\#\left\{\omega_a \in \Omega_a \cap R : \left\{\begin{smallmatrix}\bigvee_{\Omega\in\mathcal{M}} \\ \bigwedge_{\Omega\in\mathcal{M}}\end{smallmatrix}\right\} \operatorname{dist}(\omega_a, \Omega) \left\{\begin{smallmatrix}\leq \\ >\end{smallmatrix}\right\} \theta\right\}}{\#\left\{\omega_b \in \Omega_b \cap R : \left\{\begin{smallmatrix}\bigvee_{\Omega\in\mathcal{M}} \\ \bigwedge_{\Omega\in\mathcal{M}}\end{smallmatrix}\right\} \operatorname{dist}(\omega_b, \Omega) \left\{\begin{smallmatrix}\leq \\ >\end{smallmatrix}\right\} \theta\right\}}. \tag{S5}$$

Here, we use the combinations $(\Omega_a, \mathcal{M}, \Omega_b)$ listed in Table S5 where, again, the distinction 'or' or 'and' only applies for $\mathcal{M}$ being a object combination. This results in 2 (inequalitites) $\times$ $\big[$2 ('or'/'and') $\times$ 4 (for combined $\mathcal{M}$) $+$ 8 (for non-combined $\mathcal{M}$)$\big] = 32$ features per distance threshold.

In total, we thus obtain 279 features per distance threshold times 7 distance thresholds according to Equation 5 in the main article, i.e., 1953 distance-based features per tissue region, see Table S1.

Ω_a	Ω_b
$CD4^{any}$	Ki67
$CD4^{prolif.}$	Ki67
$CD4^{non\text{-}prolif.}$	Ki67
$CD8^{any}$	Ki67
$CD8^{prolif.}$	Ki67
$CD8^{non\text{-}prolif.}$	Ki67
$CD4^{any} \cup CD8^{any}$	Ki67
$CD4^{prolif.} \cup CD8^{prolif.}$	Ki67
$CD4^{non\text{-}prolif.} \cup CD8^{non\text{-}prolif.}$	Ki67
Ki67	$CD4^{prolif.}$
Ki67	$CD4^{non\text{-}prolif.}$
Ki67	$CD8^{prolif.}$
Ki67	$CD8^{non\text{-}prolif.}$

Table S2. Object combinations for distance-based fractions.

Ω_a	$\mathcal{M}$
$CD4^{any}$	$\{CD8^{prolif.}, CD8^{non\text{-}prolif.}\}$
$CD4^{prolif.}$	$\{CD8^{prolif.}, CD8^{non\text{-}prolif.}\}$
$CD4^{prolif.}$	$\{CD8^{prolif.}, Ki67\}$
$CD4^{prolif.}$	$\{CD4^{non\text{-}prolif.}, CD8^{non\text{-}prolif.}\}$
$CD4^{non\text{-}prolif.}$	$\{CD8^{prolif.}, CD8^{non\text{-}prolif.}\}$
$CD4^{non\text{-}prolif.}$	$\{CD8^{prolif.}, Ki67\}$
$CD4^{non\text{-}prolif.}$	$\{CD4^{prolif.}, CD8^{prolif.}, Ki67\}$
$CD8^{any}$	$\{CD4^{prolif.}, CD4^{non\text{-}prolif.}\}$
$CD8^{prolif.}$	$\{CD4^{prolif.}, CD4^{non\text{-}prolif.}\}$
$CD8^{prolif.}$	$\{CD4^{prolif.}, Ki67\}$
$CD8^{prolif.}$	$\{CD4^{non\text{-}prolif.}, CD8^{non\text{-}prolif.}\}$
$CD8^{non\text{-}prolif.}$	$\{CD4^{prolif.}, CD4^{non\text{-}prolif.}\}$
$CD8^{non\text{-}prolif.}$	$\{CD4^{prolif.}, Ki67\}$
$CD8^{non\text{-}prolif.}$	$\{CD4^{prolif.}, CD8^{prolif.}, Ki67\}$
Ki67	$\{CD4^{prolif.}, CD4^{non\text{-}prolif.}\}$
Ki67	$\{CD8^{prolif.}, CD8^{non\text{-}prolif.}\}$
Ki67	$\{CD4^{prolif.}, CD4^{non\text{-}prolif.}, CD8^{prolif.}, CD8^{non\text{-}prolif.}\}$
Ki67	$\{CD4^{prolif.}, CD8^{prolif.}\}$
Ki67	$\{CD4^{non\text{-}prolif.}, CD8^{non\text{-}prolif.}\}$

Table S3. Object combinations for distance-based ratios.

Ω_a	$\mathcal{M}$	Ω_b
Ki67	$\{CD8^{prolif.}, CD8^{non\text{-}prolif.}\}$	$CD4^{any}$
Ki67	$\{CD8^{prolif.}\}$	$CD4^{prolif.}$
Ki67	$\{CD8^{non\text{-}prolif.}\}$	$CD4^{non\text{-}prolif.}$
Ki67	$\{CD4^{prolif.}, CD4^{non\text{-}prolif.}\}$	$CD8^{any}$
Ki67	$\{CD4^{prolif.}\}$	$CD8^{prolif.}$
Ki67	$\{CD4^{non\text{-}prolif.}\}$	$CD8^{non\text{-}prolif.}$
$CD4^{any}$	$\{CD8^{prolif.}, CD8^{non\text{-}prolif.}\}$	Ki67
$CD4^{prolif.}$	$\{CD8^{prolif.}\}$	Ki67
$CD4^{non\text{-}prolif.}$	$\{CD8^{non\text{-}prolif.}\}$	Ki67
$CD8^{any}$	$\{CD4^{prolif.}, CD4^{non\text{-}prolif.}\}$	Ki67
$CD8^{prolif.}$	$\{CD4^{prolif.}\}$	Ki67
$CD8^{non\text{-}prolif.}$	$\{CD4^{non\text{-}prolif.}\}$	Ki67
$CD4^{prolif.}$	$\{Ki67\}$	$CD4^{any}$
$CD4^{non\text{-}prolif.}$	$\{Ki67\}$	$CD4^{any}$
$CD8^{prolif.}$	$\{Ki67\}$	$CD8^{any}$
$CD8^{non\text{-}prolif.}$	$\{Ki67\}$	$CD8^{any}$
$CD4^{prolif.}$	$\{Ki67\}$	$CD4^{any} \cup CD8^{any}$
$CD4^{non\text{-}prolif.}$	$\{Ki67\}$	$CD4^{any} \cup CD8^{any}$
$CD8^{prolif.}$	$\{Ki67\}$	$CD4^{any} \cup CD8^{any}$
$CD8^{non\text{-}prolif.}$	$\{Ki67\}$	$CD4^{any} \cup CD8^{any}$
$CD4^{prolif.} \cup CD8^{prolif.}$	$\{Ki67\}$	$CD4^{any} \cup CD8^{any}$
$CD4^{non\text{-}prolif.} \cup CD8^{non\text{-}prolif.}$	$\{Ki67\}$	$CD4^{any} \cup CD8^{any}$

Table S4. Object combinations for ratios with distance criterion in the numerator only.

Ω_a	$\mathcal{M}$	Ω_b
Ki67	$\{CD8^{prolif.}, CD8^{non\text{-}prolif.}\}$	$CD4^{any}$
Ki67	$\{CD8^{prolif.}\}$	$CD4^{prolif.}$
Ki67	$\{CD8^{non\text{-}prolif.}\}$	$CD4^{non\text{-}prolif.}$
Ki67	$\{CD4^{prolif.}, CD4^{non\text{-}prolif.}\}$	$CD8^{any}$
Ki67	$\{CD4^{prolif.}\}$	$CD8^{prolif.}$
Ki67	$\{CD4^{non\text{-}prolif.}\}$	$CD8^{non\text{-}prolif.}$
$CD4^{any}$	$\{CD8^{prolif.}, CD8^{non\text{-}prolif.}\}$	Ki67
$CD4^{prolif.}$	$\{CD8^{prolif.}\}$	Ki67
$CD4^{non\text{-}prolif.}$	$\{CD8^{non\text{-}prolif.}\}$	Ki67
$CD8^{any}$	$\{CD4^{prolif.}, CD4^{non\text{-}prolif.}\}$	Ki67
$CD8^{prolif.}$	$\{CD4^{prolif.}\}$	Ki67
$CD8^{non\text{-}prolif.}$	$\{CD4^{non\text{-}prolif.}\}$	Ki67

Table S5. Object combinations for ratios with distance criterion in both numerator and denominator. These combinations are a subset of those listed in Table S4.

2 DETAILS ON DISCRIMINATORY FEATURES FOR THE MSI/MSS DATASET

The five features shown in Figure 5 in the main article were selected from the 20 features with largest OPM values. Figures S1, S2, and S3 show information about all these 20 features.

OPM	Rank		ROC-AUC	# MSI	# MSS
**0.788**	1	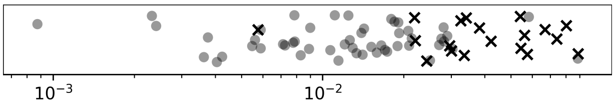	0.902	19	52
		$\frac{\#\{\omega \in \text{Ki67} \cap \text{tumor} : \text{dist}(\omega, \text{CD4}^{\text{non-prolif.}}) \leq 15\,\mu\text{m} \vee \text{dist}(\omega, \text{CD8}^{\text{non-prolif.}}) \leq 15\,\mu\text{m}\}}{\#(\text{Ki67} \cap \text{tumor})}$			
**0.778**	2	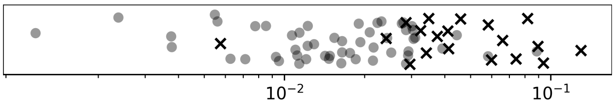	0.889	19	53
		$\frac{\#\{\omega \in \text{Ki67} : \text{dist}(\omega, \text{CD4}^{\text{non-prolif.}}) \leq 15\,\mu\text{m} \vee \text{dist}(\omega, \text{CD8}^{\text{non-prolif.}}) \leq 15\,\mu\text{m}\}}{\#(\text{Ki67})}$			
**0.757**	3	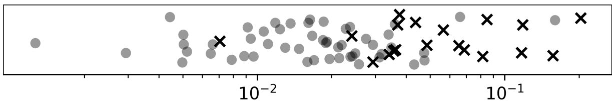	0.886	19	52
		$\frac{\#\{\omega \in \text{Ki67} \cap \text{tumor} : \text{dist}(\omega, \text{CD4}^{\text{any}}) \leq 15\,\mu\text{m} \vee \text{dist}(\omega, \text{CD8}^{\text{any}}) \leq 15\,\mu\text{m}\}}{\#(\text{Ki67} \cap \text{tumor})}$			
**0.755**	4	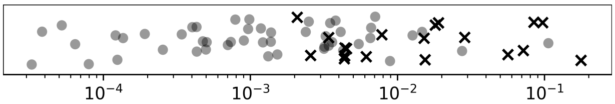	0.885	19	52
		$\frac{\#\{\omega \in \text{CD8}^{\text{non-prolif.}} \cap \text{tumor} : \text{dist}(\omega, \text{CD4}^{\text{non-prolif.}}) > 100\,\mu\text{m}\}}{\#\{\omega \in \text{Ki67} \cap \text{tumor} : \text{dist}(\omega, \text{CD4}^{\text{non-prolif.}}) > 100\,\mu\text{m}\}}$			
**0.749**	5	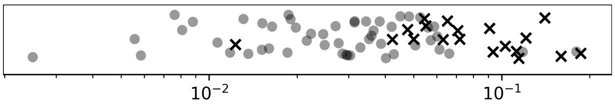	0.882	19	52
		$\frac{\#\{\omega \in \text{Ki67} \cap \text{tumor} : \text{dist}(\omega, \text{CD4}^{\text{non-prolif.}}) \leq 20\,\mu\text{m} \vee \text{dist}(\omega, \text{CD8}^{\text{non-prolif.}}) \leq 20\,\mu\text{m}\}}{\#(\text{Ki67} \cap \text{tumor})}$			
**0.734**	6	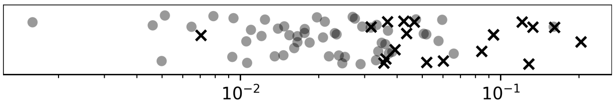	0.867	19	53
		$\frac{\#\{\omega \in \text{Ki67} : \text{dist}(\omega, \text{CD4}^{\text{any}}) \leq 15\,\mu\text{m} \vee \text{dist}(\omega, \text{CD8}^{\text{any}}) \leq 15\,\mu\text{m}\}}{\#(\text{Ki67})}$			
**0.727**	7	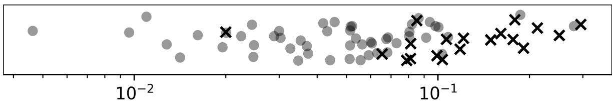	0.870	19	52
		$\frac{\#\{\omega \in \text{Ki67} \cap \text{tumor} : \text{dist}(\omega, \text{CD4}^{\text{non-prolif.}}) \leq 25\,\mu\text{m} \vee \text{dist}(\omega, \text{CD8}^{\text{non-prolif.}}) \leq 25\,\mu\text{m}\}}{\#(\text{Ki67} \cap \text{tumor})}$			

Figure S1. Features ranked by OPM value. Ranks 1 to 7.

OPM	Rank		ROC-AUC	# MSI	# MSS
**0.726**	8	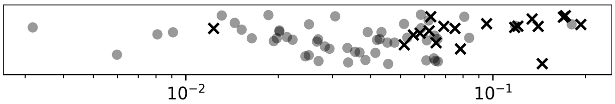	0.863	19	53
		$\frac{\#\{\omega \in \text{Ki67} : \text{dist}(\omega, \text{CD4}^{\text{non-prolif.}}) \leq 20\,\mu\text{m} \vee \text{dist}(\omega, \text{CD8}^{\text{non-prolif.}}) \leq 20\,\mu\text{m}\}}{\#(\text{Ki67})}$			
**0.725**	9	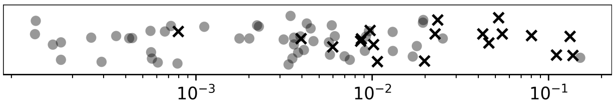	0.869	19	52
		$\frac{\#\{\omega \in \text{Ki67} \cap \text{tumor} : \text{dist}(\omega, \text{CD8}^{\text{non-prolif.}}) \leq 20\,\mu\text{m}\}}{\#(\text{Ki67} \cap \text{tumor})}$			
**0.725**		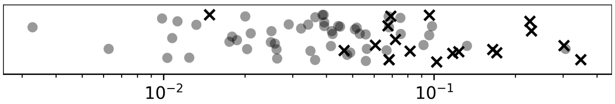	0.869	19	52
		$\frac{\#\{\omega \in \text{Ki67} \cap \text{tumor} : \text{dist}(\omega, \text{CD4}^{\text{any}}) \leq 20\,\mu\text{m} \vee \text{dist}(\omega, \text{CD8}^{\text{any}}) \leq 20\,\mu\text{m}\}}{\#(\text{Ki67} \cap \text{tumor})}$			
**0.711**	11	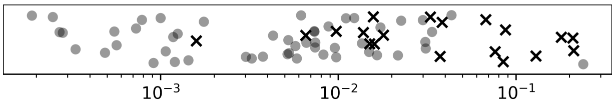	0.862	19	52
		$\frac{\#\{\omega \in \text{Ki67} \cap \text{tumor} : \text{dist}(\omega, \text{CD8}^{\text{non-prolif.}}) \leq 25\,\mu\text{m}\}}{\#(\text{Ki67} \cap \text{tumor})}$			
**0.710**	12	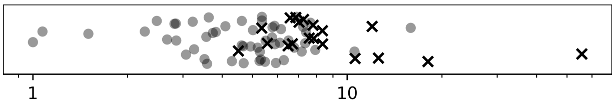	0.855	19	53
		$\underset{R \in \{1000\,\mu\text{m tiles}\}}{\text{rel. perc. 97}} \frac{\#\{\omega \in \text{CD4}^{\text{non-prolif.}} \cap R : \text{dist}(\omega, \text{Ki67}) \leq 20\,\mu\text{m}\}}{\#\{\omega \in (\text{CD8}^{\text{any}}) \cap R : \text{dist}(\omega, \text{Ki67}) \leq 20\,\mu\text{m}\}}$			
**0.710**		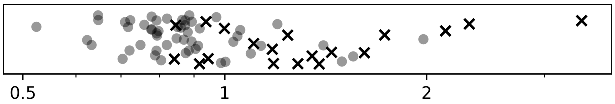	0.855	19	53
		$\underset{R \in \{500\,\mu\text{m tiles}\}}{\text{COV}} \frac{\#\{\omega \in (\text{CD4}^{\text{any}}) \cap R : \text{dist}(\omega, \text{Ki67}) \leq 35\,\mu\text{m}\}}{\#\{\omega \in (\text{CD4}^{\text{prolif.}} \cup \text{CD8}^{\text{prolif.}}) \cap R : \text{dist}(\omega, \text{Ki67}) \leq 35\,\mu\text{m}\}}$			
**0.709**	14	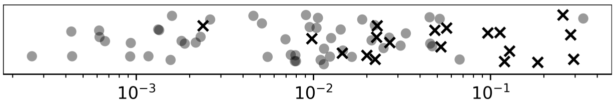	0.861	19	52
		$\frac{\#\{\omega \in \text{Ki67} \cap \text{tumor} : \text{dist}(\omega, \text{CD8}^{\text{non-prolif.}}) \leq 30\,\mu\text{m}\}}{\#(\text{Ki67} \cap \text{tumor})}$			

Figure S2. Features ranked by OPM value. Ranks 8 to 14.

OPM	Rank		ROC-AUC	# MSI	# MSS
**0.708**	15	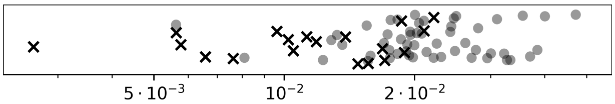 $\frac{\#\{\omega \in \mathrm{CD4}^{\text{prolif.}} : \mathrm{dist}(\omega, \mathrm{Ki67}) > 50\,\mu\mathrm{m}\}}{\#(\mathrm{CD4}^{\text{any}} \cup \mathrm{CD8}^{\text{any}})}$	0.854	19	53
**0.708**		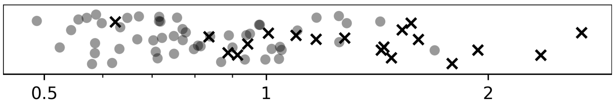 $\underset{R\in\{1000\,\mu\mathrm{m\ tiles}\}}{\mathrm{COV}} \frac{\#\{\omega \in \mathrm{CD4}^{\text{any}} \cap R : \mathrm{dist}(\omega, \mathrm{Ki67}) \leq 30\,\mu\mathrm{m}\}}{\#\{\omega \in (\mathrm{CD4}^{\text{prolif.}} \cup \mathrm{CD8}^{\text{prolif.}}) \cap R : \mathrm{dist}(\omega, \mathrm{Ki67}) \leq 30\,\mu\mathrm{m}\}}$	0.854	19	53
**0.708**		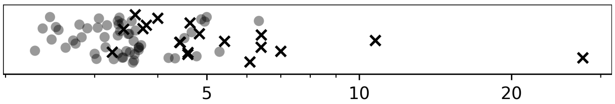 $\underset{R\in\{500\,\mu\mathrm{m\ tiles}\}}{\text{rel. perc. } 95} \frac{\#\{\omega \in \mathrm{CD4}^{\text{any}} \cap R : \mathrm{dist}(\omega, \mathrm{Ki67}) \leq 35\,\mu\mathrm{m}\}}{\#\{\omega \in (\mathrm{CD4}^{\text{prolif.}} \cup \mathrm{CD8}^{\text{prolif.}}) \cap R : \mathrm{dist}(\omega, \mathrm{Ki67}) \leq 35\,\mu\mathrm{m}\}}$	0.854	19	53
**0.705**	18	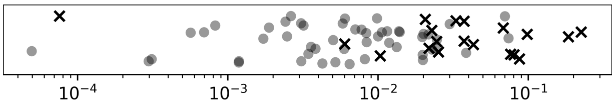 $\frac{\#\{\omega \in \mathrm{CD8}^{\text{non-prolif.}} \cap \text{tumor} : \mathrm{dist}(\omega, \mathrm{Ki67}) \leq 15\,\mu\mathrm{m}\}}{\#((\mathrm{CD4}^{\text{any}} \cup \mathrm{CD8}^{\text{any}}) \cap \text{tumor})}$	0.859	19	52
**0.703**	19	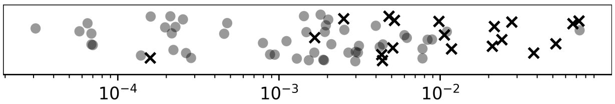 $\frac{\#\{\omega \in \mathrm{Ki67} \cap \text{tumor} : \mathrm{dist}(\omega, \mathrm{CD8}^{\text{non-prolif.}}) \leq 15\,\mu\mathrm{m}\}}{\#(\mathrm{Ki67} \cap \text{tumor})}$	0.858	19	52
**0.702**	20	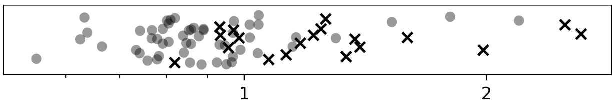 $\underset{R\in\{500\,\mu\mathrm{m\ tiles}\}}{\mathrm{COV}} \frac{\#\{\omega \in \mathrm{CD4}^{\text{any}} \cap R : \mathrm{dist}(\omega, \mathrm{Ki67}) \leq 30\,\mu\mathrm{m}\}}{\#\{\omega \in (\mathrm{CD4}^{\text{prolif.}} \cup \mathrm{CD8}^{\text{prolif.}}) \cap R : \mathrm{dist}(\omega, \mathrm{Ki67}) \leq 30\,\mu\mathrm{m}\}}$	0.851	19	53

Figure S3. Features ranked by OPM value. Ranks 15 to 20.
